# Is Support for Feminism Enough for Change? How Sexism and Gender Stereotypes Might Hinder Gender Justice

**DOI:** 10.3389/fpsyg.2022.912941

**Published:** 2022-07-12

**Authors:** Gloria Jiménez-Moya, Héctor Carvacho, Belén Álvarez, Camila Contreras, Roberto González

**Affiliations:** ^1^Escuela de Psicología, Pontificia Universidad Católica de Chile, Santiago, Chile; ^2^School of Psychology, The University of Queensland, Brisbane, QLD, Australia

**Keywords:** sexism, gender stereotypes, feminist movement, social justice, latent profile analyses

## Abstract

Even though formal processes (i.e., gender quotes) are necessary to achieve gender justice, attitudinal changes (i.e., support of egalitarian social norms) are also essential. The endorsement of sexism and gender stereotypes perpetuate inequality on a daily basis, and can be seen as barriers that prevent societies from reaching social justice. Therefore, changing sexist social norms can be understood as a fundamental step in accomplishing gender justice. With the aim of studying Chileans’ sexist norms, we conducted a survey with a representative sample (*N* = 490) exploring levels of sexism and gender stereotypes, as well as support for the feminist movement. Using Latent Profile Analysis, we identified four groups of citizens: (1) a first group that shows high levels of sexism and low support for the feminist movement (9%); (2) a second group, with low levels of sexism and high support for the feminist movement (20%); (3) a third group with high levels of sexism and high support for the feminist movement (65%); and (4) a fourth group with mid-levels of sexism and support of the feminist movement (6%). We called these groups the Sexist, Feminist, Inconsistent, and Moderate Group, respectively. The four groups showed similar high endorsement of gender stereotypes. These results are twofold. First, they hint that although nowadays gender equality seems to be generally accepted, this coexists with a high prevalence of sexist social norms, represented by the inconsistent group being the most prevalent. Second, gender stereotypes are still deeply rooted in Chilean culture, surprisingly even among feminist citizens.

## Introduction

Chile is currently experiencing unprecedented social changes in intergroup relations. One of the main changes relates to gender inequality, where important achievements have been made in the last years, presumably as a consequence of the visibility of a strong feminist movement (see Comunidad Mujer, 2018). For instance, in the context of the ongoing constitutional process, Chilean citizens voted in October 2020 to have a gender parity Constitutional Assembly, the panel in charge of drafting a new constitution. This implies that the constitutional text would be drafted by a similar number of men and women, which was globally unprecedented.

However, these legal or official enhancements do not necessarily lead to social justice. Sexist social norms, namely sexism and gender stereotypes, might still prevail regardless of top-down changes such as equality laws. We argue that changing social gender norms is also a fundamental step in accomplishing social justice for women.

The aim of this work is to study Chileans’ social norms regarding gender inequality, namely hostile and benevolent sexism, support for traditional gender stereotypes and support for the feminist movement. We argue that understanding individuals’ attitudes will lead to suitable interventions to build social justice, not only based on top-down changes, but also from the bottom-up.

## How to Approach Gender Justice

Justice is a necessary condition to live in a dignified way. The lack of social justice affects disadvantaged groups through prejudice and inequality (see Hatzenbuehler et al., 2013; Lewis et al., 2015; Rosenthal, 2016) by maintaining their low status and their lack of resources, resulting in the perpetuation of discrimination.

When it comes to gender inequality, sexist experiences diminish women’s perception that the world is a just place for them, which in turn predicts lower levels of personal control and mental health issues (Fischer and Bolton Holz, 2010). Based on this, sexist discrimination can be framed as a violation of women’s human rights, and understood as a social justice issue (see Stewart and Zucker, 2016).

In order to achieve gender justice, different strategies need to be applied. Institutional support emerges as a determinant factor to approach social change (Allport, 1954); thus gender-sensitive laws are critical in building egalitarian societies (see Terjesen et al., 2015). Nevertheless, structural changes do not necessarily imply social change at every level. For instance, although in Chile the law allows fathers to take paternity leave after their child’s birth, only 0.2% of men decide to take it (Superintendencia de Seguridad Social, 2016). This might relate to the fact that men who take a longer paternity leave are perceived more negatively than those who take a shorter leave (Gartzia et al., 2018), or because the family income level could be negatively affected (Duvander et al., 2021).

Therefore, top-down changes that come from institutions do not undoubtedly lead to social change. We argue that bottom-up changes are also necessary to trigger real evolution in individuals’ behavior. That is to say, changes that are not imposed by authorities but built in the daily context of intergroup relations also affect behavioral tendencies. In fact, social norms are understood as vehicles of social change that are useful in generating more egalitarian contexts (Tankard and Paluck, 2016; Prentice and Paluck, 2020). In this sense, sexist social norms that justify and legitimize gender inequality might impede social justice for women, in the same way that racism obstructs justice (see Caldwell and Bledsoe, 2019).

## The Never-Ending Story: Sexism and Gender Stereotypes

Sexism can be expressed through different means. Glick and Fiske (1996) described that sexist prejudice encompasses two types of sexism that coexist: hostile and benevolent sexism. Hostile attitudes reflect a traditional type of prejudice (see Allport, 1954) that explicitly evaluates women in a negative way, and considers them inferior to men. Benevolent sexism describes women in stereotypical and restricted ways but using a paternalistic and, apparently, positive tone (Glick and Fiske, 1996), which makes it hard to identify as a form of prejudice (Barreto and Ellemers, 2005). Undoubtedly, sexist attitudes negatively impact women’s lives in multiple ways (e.g., Undurraga and López Hornickel, 2020). Sexism can impact women’s habits and health (see Rapp et al., 2021): for instance, women who experienced either hostile or benevolent sexism during the lab session in an experimental study reported consuming more alcoholic drinks later that evening compared to women who did not experience sexism (Hamilton and DeHart, 2020). This negative effect of sexism on unhealthy habits is mediated by psychological distress (Zucker and Landry, 2007).

Identity and motivational aspects are also affected by experienced sexism, as sexist teasing negatively affects gender self-esteem in women (Hack et al., 2019) and the perception of sexist barriers predicts the disparity between women’s precollege ambitions and their current attempts to continue studying after graduation (Li et al., 2021). Sexism also predicts individuals’ tolerance for sexual harassment (e.g., Hill and Marshall, 2018; Shi and Zheng, 2020), men’s victim blaming and approval of the aggressor’s behavior (Koepke et al., 2014), and how women who confront discrimination are perceived (Jiménez-Moya et al., 2022). In public domains, sexism hinders women’s goals and achievements when approaching leadership positions (Rudman et al., 2012), predicts negative attitudes toward female leaders (e.g., Good and Rudman, 2010) and discriminatory preferences for political candidates (Gervais and Hillard, 2011; Ratliff et al., 2017). In sum, sexist attitudes obstruct social justice for women.

Sexism is also about perpetuating traditional gender roles and stereotypes. Men are typically associated with agentic traits such as competence and assertiveness, while women are associated with communal attributes, such as warmth and care (see Glick and Fiske, 1999; Kite et al., 2008). Gender stereotypes not only strictly differentiate men and women, but also generate social expectations toward them and how they should behave (see Ellemers, 2018; Guerra et al., 2021), thus gender stereotyping has numerous implications. For example, female students are perceived as less talented and competent than male students in scientific fields (e.g., Leslie et al., 2015; Carli et al., 2016; Grunspan et al., 2016). Furthermore, women’s lower performance in negotiations about salary and benefits is predicted by their male counterparts’ stereotypes (Pardal et al., 2020). Gender stereotypes negatively affect women’s (and men’s) development since childhood (e.g., Brown and Stone, 2016; Bian et al., 2017), indirectly preventing social justice, as stereotyping women creates barriers for them in areas traditionally assigned to men. Gender stereotypes also directly affect social justice perceptions. Gender roles and stereotypes are complementary; that is, they prescribe both men and women with positive and negative attributes, advantages and disadvantages (Glick and Fiske, 1999; Ellemers, 2018). This complementarity between social groups stereotypes triggers the perception that the system is fair and legitimate (Kay and Jost, 2003), presumably because it shows that benefits are equally allocated. Thus, exposure to and visibility of complementary gender stereotypes and related benevolent beliefs leads women—and in some circumstances men—to support the current state of gender relations and the system in general that they wrongly perceive as fair and equitable (Jost and Kay, 2005).

A way of challenging sexism and gender stereotypes might be to support social change, namely by standing up for the feminist movement. It is well known that this movement has grown in the last few years worldwide and, as was mentioned, it has had a powerful impact in Chilean society (see Comunidad Mujer, 2018) by raising awareness of gender inequality. Thus, supporting the feminist movement can be understood as a way of opposing traditional gender views and to approach gender social justice.

## The Present Research

Sexism and gender stereotypes have negative consequences for women, as they lead to essential beliefs regarding the differences between men and women (Morton et al., 2009) and are used to justify and support unfair gender relations (Jost and Kay, 2005). We argue that, to approach social justice for women, it is crucial to study social gender norms that emerge in a daily context and hinder social justice. We argue that by knowing and understanding individuals’ attitudes we will be able to, first, be aware of the current attitudes regarding gender inequality and, second, design suitable interventions to reach gender justice. Therefore, the aim of this work is to examine Chileans’ levels of hostile and benevolent sexism and gender stereotypes, as well as their support for the feminist movement, as a proxy of their support for social justice. To accomplish this, we used latent profile analysis (LPA), which allows us to identify different profiles of individuals according to their attitudes; as such, this method is more appropriate to reach our aim than other research analyses. Traditional research methods are variable-centered, where variables are treated as the unit of analysis, and it is assumed that the associations between variables are consistent across the population (Collins and Lanza, 2009; Osborne and Sibley, 2017). LPA, on the other hand, is an analytic method that is person-centered, meaning the person is the unit of analysis. This type of approach allows us to identify subgroups of people who share certain characteristics and who respond to critical measures in a certain way that differentiate them from other groups of people (Osborne and Sibley, 2017). By using LPA, we are better positioned to understand the different groups of people that emerge in Chilean society, based on their social gender norms.

In addition, we were also interested in studying other attitudes that might help to better understand the different profiles that might emerge. We argue that social gender norms are related to a larger set of beliefs regarding equality and social justice in general. In fact, those who report sexist gender norms are also more prone to endorse conservative ideologies that perpetuate social inequality (Figueiredo et al., 2017). Thus, we also include ideological attitudes in our analyses, namely perception of economic inequality, social dominance orientation and right-wing authoritarianism, as these might be relevant in the study of gender inequality (see Sidanius and Pratto, 1999; Brandt and Henry, 2012; Figueiredo et al., 2017; Kteily et al., 2017; Ratliff et al., 2017). These variables were used as predictors of profile memberships, to explore in more depth how profiles differ from each other (Osborne and Sibley, 2017). That is, once we have our latent profiles, these variables will be included in a multinomial regression model to assess the likelihood of being assigned to one of the profiles, depending on the participants’ ideological attitudes (Asparouhov and Muthén, 2014).

## Materials and Methods

### Participants and Procedure

The data for this study was part of a survey conducted by MIDE UC Measurement Center at Pontificia Universidad Católica de Chile, aimed at studying Chileans’ perceptions regarding certain social issues. Data was collected between September 2019 and March 2020 using a randomized and stratified sampling. The sample was representative of the adult population living in the country’s five largest urban areas and was composed of 490 Chileans (38.4% men, 61.6% women), between 18 and 69 years of age (*M*_Age_ = 42.7, SD = 14.47).

Data was collected using computer-assisted personal interviews at the respondents’ home addresses. The survey took about 60 min to complete. This study was approved by the ethics committee at Pontificia Universidad Católica in Chile and all participants signed an informed consent form.

### Measurements

*Hostile and benevolent sexism*. We used a selection of 8 items from the *Ambivalent Sexism Inventory* (Glick and Fiske, 1996) adapted into Spanish by Cárdenas et al. (2010). Four items measured hostile sexism (e.g., “In the name of equality, many women try to get certain privileges,” *α* = 0.66) and four items measured benevolent sexism (e.g., “No matter how accomplished he is, a man is not truly complete as a person unless he has the love of a woman,” *α* = 0.77; a full list of items can be found in the Supplementary Material). Participants indicated their agreement with each statement using a scale ranging from 1 (*Strongly disagree*) to 5 (*Strongly agree*).

*Traditional gender stereotypes.* To measure gender stereotypes, we asked participants to read a list with 7 traits and indicate using a 1 (*Very uncharacteristic*) to 5 (*Highly characteristic*) scale, to what extend those traits are characteristics of a typical man or woman. For each of the given target group (man or woman), two subscales were created: One taping agentic traits (ambition, superior intelligence, self-confidence and independence) and the other communal traits (kindness, cooperation, and good listener; see Glick et al., 2000; Ellemers, 2018).

A confirmatory factor analysis using Mplus 8.0 (Muthén and Muthén, 1998–2017) confirmed for man the existence of the two subscales one regarding the agentic traits, most commonly associated with masculine stereotypes (*α* = 0.72) and one for communal traits, most commonly associated with feminine stereotypes (*α* = 0.86). This analysis confirmed that both subscales had an acceptable fit [*X*^2^(13) = 100.940, *p* = 0.068; RMSEA = 0.083; CFI = 0.969; SRMR = 0.032].

With regard to the feminine stereotypes, the confirmatory factor analysis also reveals the existence of the agentic (*α* = 0.79) and the communal (*α* = 0.83) traits subscales, and that both of them had an acceptable fit [*X*^2^(13) = 82.187, *p* = 0.059; RMSEA = 0.074; CFI = 0.975; SRMR = 0.025].

In the subsequent Latent Profile Analysis, we include only the agentic traits subscale (stereotypically masculine), obtained from the measure of a “typical man,” and the communal traits subscale for women (stereotypically feminine), obtained from the measure of a “typical woman” (see Supplementary Material for factor loadings of masculine and feminine stereotype analyses).

*Support for feminist movement.* We created three items to measure participants’ support for the feminist movement (“I think the feminist movement is necessary today;” “I value the feminist movement in a positive way;” “I agree with the demands of the feminist movement” *α* = 0.90). Participants indicated their agreement with each statement using a scale ranging from 1 (*Strongly disagree*) to 5 (*Strongly agree*).

*Perception of economic inequality.* We measured perception of economic inequality with 1 item: “Compared to other countries in South America, where do you think Chile stands in terms of economic inequality?” Participants answered using a scale from 1 (*It is the country with lowest levels of economic inequality*) to 10 (*It is the country with highest levels of economic inequality*).

*Social Dominance Orientation.* We used four items adapted from Ho et al. (2015); e.g., “In an ideal society, some groups should be on top and others should be on the bottom,” *α* = 0.65. Participants indicated their agreement to them using a scale ranging from 1 (*Strongly disagree*) to 5 (*Strongly agree*).

*Right-Wing Authoritarianism.* We used six items adapted from Funke (2005); e.g., “What our country needs is a strong authority with the determination to set us on the right path,” *α* = 0.84. Participants indicated their agreement to them using a scale ranging from 1 (*Strongly disagree*) to 5 (*Strongly agree*).

## Results

### Latent Profile Analysis

**Figure 1:**
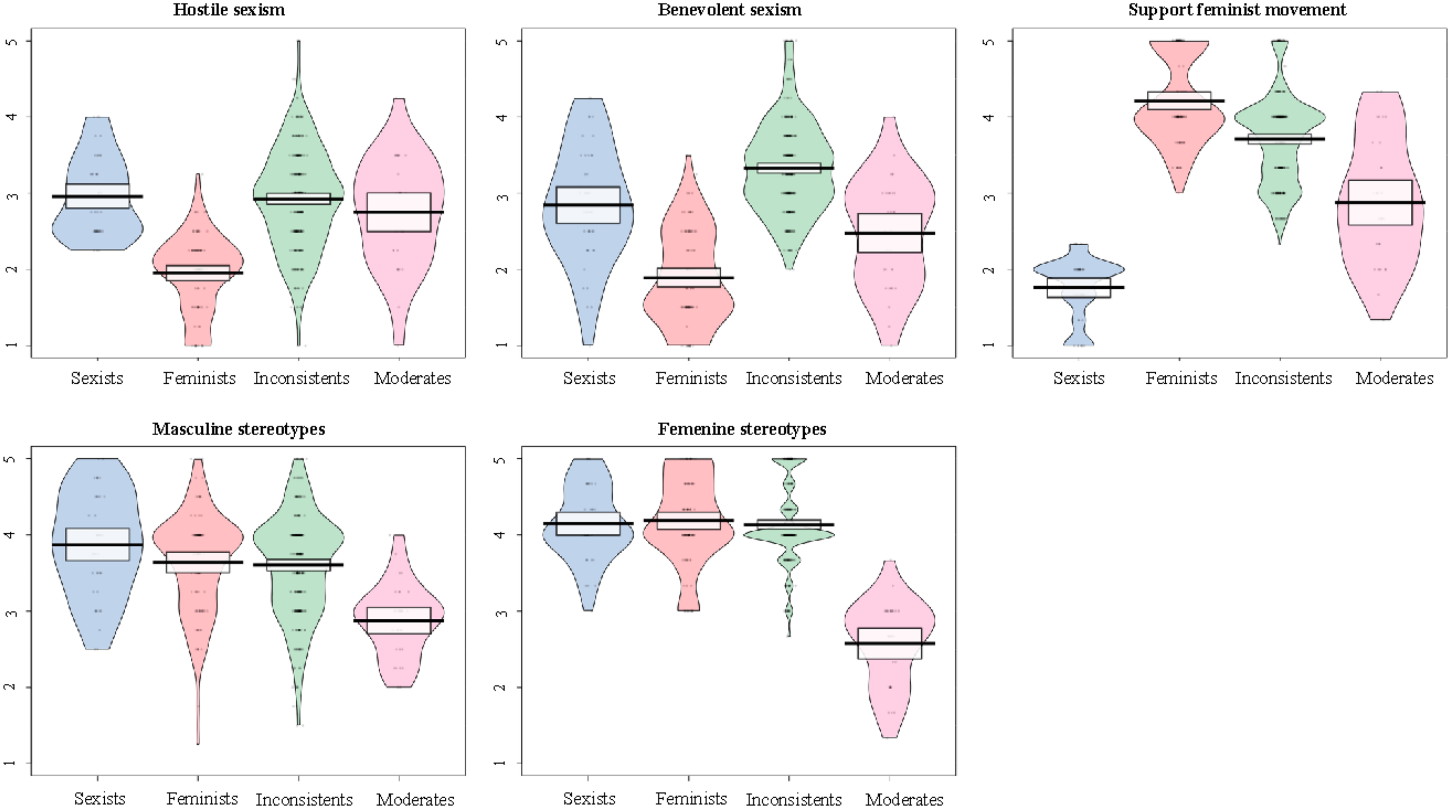
Pirate plots showing the distribution for each of the four profiles (sexists, feminists, inconsistents, and moderates) for each of the indicators of profile membership. Plots built with “yarrr” package (Phillips, 2017) in R v 4.1.2 (R Core Team, 2021). In the plots, the horizontal line is the mean, the band (rectangle) shows the 95% confidence intervals, the bean indicates the density of the data and the dots are individual data points.

Descriptive statistics and bivariate correlations are shown in Table 1. We estimated a LPA using Mplus 8.0 (Muthén and Muthén, 1998–2017) on five continuous variables: hostile sexism, benevolent sexism, support for the feminist movement, masculine stereotypes and feminine stereotypes. Following recommendations (Collins and Lanza, 2009; Asparouhov and Muthén, 2012; Osborne and Sibley, 2017), we used several indicators to assess model fit and, on this basis, decided that a four-profile solution provided the best fit (AIC = 5376.11; BIC = 5493.55; aBIC = 5404.681; LMR = 60.565, *p* = 0.096; Entropy = 0.765; see Supplementary Material for model fit solutions between one and four profiles). Figure 1 provides information about participant distribution, density, mean and confidence intervals of each of the indicators (hostile sexism, benevolent sexism, support for the feminist movement, masculine stereotypes and feminine stereotypes) across the four latent profiles membership.

The first profile, which accounts for 9% of the participants, exhibited high levels of both hostile and benevolent sexism, as well as a high rating of both masculine and feminine stereotypes. On the other hand, their support for the feminist movement was very low. We labeled this profile as the “Sexist Group.” The second profile had the lowest mean in both hostile and benevolent sexism compared to the rest of the profiles, and participants exhibited high support for the feminist movement. However, the mean for masculine and feminine stereotypes was also very high in this group, similar to the other profiles. We labeled this group as the “Feminist Group,” accounting for 20% of the sample. The third profile also exhibited high support for the feminist movement and a high rating for masculine and feminine stereotypes; however, this profile also exhibited high levels of hostile and benevolent sexism. We labeled this profile as the “Inconsistent Group,” encompassing 65% of the sample. The fourth profile accounted for 6% of the sample and was labeled the “Moderate Group,” as participants assigned to this profile had ratings close to the mean for all measures.

**Table 1:** Descriptive statistics and bivariate correlations.

Variable	Range	*M*	SD	1	2	3	4	5	6	7	8
1. Hostile sexism	1–5	2.72	0.73	–							
2. Benevolent sexism	1–5	2.94	0.85	0.41^**^	–						
3. Support feminist movement	1–5	3.59	0.87	−0.26^**^	0.01	–					
4. Masculine stereotypes	1–5	3.59	0.69	0.04	0.03	−0.03	–				
5. Feminine stereotypes	1–5	4.04	0.66	−0.02	0.12^*^	0.12^**^	0.34^**^	–			
6. Perception of inequality	1–10	7.08	2.44	−0.16^**^	−0.17^**^	0.14^**^	0.12^**^	0.03	–		
7. SDO	1–5	2.21	0.65	0.34^**^	0.26^**^	−0.19^**^	−0.01	0.04	−0.16^**^	–	
8. RWA	1–5	3.46	0.74	0.23^**^	0.31^**^	−0.19^**^	0.12^**^	0.08	−0.15^**^	0.31^**^	–

* *p* *< 0.05, and*

** *p < 0.01*.

### Latent Profile Predictors

We tested the predictors of the latent profiles using multinomial logistic regression without treating the subgroups as observed variables (Asparouhov and Muthén, 2014).

Table 2 presents results for the three-step multinomial logistic regression model assessing perception of inequality, SDO and RWA as predictors of the latent profiles. The Feminist Group was used as the reference group. This means that all results describe the likelihood of belonging to a given profile relative to the Feminist Group (to view results using other profiles as the reference group, see Supplementary Material).

**Table 2:** Multinomial logistic regression predicting profile membership using the feminist group as the reference group.

	Sexist group	Inconsistent group	Moderate group
Perception of inequality	−0.46^**^	−0.39^**^	−0.52^**^
Social dominance orientation (SDO)	2.09^**^	2.37^**^	1.99^**^
Right-wing authoritarianism (RWA)	2.13^**^	2.00^**^	0.58

*A negative coefficient increases the likelihood of belonging to the reference group, e.g., having high perception of inequality increases the likelihood of being assigned to the feminist profile (vs. sexist profile)*.

** *p < 0.01*.

As can be seen in Table 2, perceiving higher economic inequality predicts membership to the Feminist Group (vs. Sexist, Inconsistent and Moderate groups). Participants who belong to the Feminist Group (who, we assume, perceive greater levels of gender inequality) perceive the greatest levels of economic inequality. Higher scores in SDO predicted membership to the Sexist, Inconsistent and Moderate groups (vs. Feminist group). This is consistent with previous research, as we can expect that people who have a strong social dominance orientation will justify power differences between men and women (see Sidanius and Pratto, 1999; Figueiredo et al., 2017; Sidanius et al., 2017; Schmitz et al., 2021) and hence will not be assigned to the Feminist Group. We found a similar effect using RWA as a predictor for profile membership. Higher scores on RWA predicted membership to the Sexist and Inconsistent groups (vs. feminists), but not for the Moderate Group. This is also consistent with RWA research, as people with higher levels of authoritarianism also want to preserve the structure of society as it once was (see Altemeyer, 1981; Carvacho et al., 2013; Süssenbach and Carvacho, 2021).

## Discussion

The aim of this work was to study Chileans’ gender norms, namely hostile and benevolent sexism, support for traditional gender stereotypes and support for the feminist movement. A LPA showed four types of groups of individuals, according to their social gender norms. Three of these groups presented consistent gender norms. The Feminist Group (20%) presented low levels of both hostile and benevolent sexism, and the highest support for the feminist movement, whereas individuals assigned to the Sexist Group (9%) showed high levels of sexism and the lowest support for the feminist movement. The Moderate Group (6%) reported average levels for all variables, thus it might be composed of individuals who do not have strong or well-defined norms or were simply not properly motivated to participate in the study. The vast majority of our participants (65%) were assigned to the Inconsistent Group, who presented mixed norms. They showed high levels of sexism—even higher than the Sexist Group—especially for benevolent sexism, but they also seemed to support the feminist movement. Thus, a large part of the sample shows contradictory norms regarding gender equality. This might be showing that nowadays two conflicting social norms are present in Chilean society: to support gender equality while also maintaining traditional gender relations. Although individuals appear motivated to support social change, which is concurrent with the growing social protests that are taking place worldwide (e.g., McKane and McCammon, 2018; BBC, 2020; Glas and Spierings, 2020), it may be that they are conforming to the norm of equality for external reasons, such as social pressure to be seen as an egalitarian in a context where equality is increasingly validated and approved. This might imply that, although individuals report they support feminism, they are not truly embracing its moral principles or behaving accordingly with the daily changes that the movement demands. However, it might also be that individuals do think sexism—and especially benevolent sexism—reflects real positive attitudes toward women that benefit them (see Barreto and Ellemers, 2015). In fact, men who show high levels of benevolent sexism tend to confront sexism, but based on paternalistic reasons (Estevan-Reina et al., 2020). Therefore, paradoxically, an apparent behavior to improve social justice for women (i.e., confronting sexism) contributes to perpetuating the status quo. This paradox is also present in certain Chilean public policies aimed at improving women’s living conditions, but rooted in traditional gender stereotypes, contradictory notions and homogenizing views of women (see Murray, 2013; Gideon et al., 2021; Murray and Tapia, 2021).

All profiles showed similar support for traditional gender stereotypes. This shows the resistance of gender stereotypes (see Greenwald and Banaji, 1995; Amodio and Cikara, 2021) and the fact that even feminist individuals endorse traditional gender views. These traditional stereotypes are particularly resistant in Latin America due to the presence of gender roles and ideologies rooted in Catholicism, such as *Marianismo*, the idea that Mary, the mother of Jesus, is an example of obedience and maternity for women, and *Machismo*, the stereotypical view of men as brave fighters such as the Spanish conquerors or Indigenous warriors (see González et al., 2016).

In a context where a seeming support for the feminist movement coexists with sexist beliefs, and where gender stereotypes remain, social justice for women is not plausible. Based on these results, we argue that it is necessary to design and apply interventions with a social justice perspective (see Schwartz and Lindley, 2008) aimed at creating new gender norms which establish (1) that paternalistic and benevolent attitudes have negative consequences for women and (2) the need to make individuals aware of automatic sexist bias, even among egalitarian people. For instance, in Chile school interventions have aimed at reducing stereotypes by providing tools for adolescents to increase awareness about their own endorsement of stereotypes (see Luengo Kanacri and Jiménez-Moya, 2017).

Results also show that ideological variables are related to the profiles described. First, perceiving a higher economic inequality increased the likelihood of belonging to the Feminist Group. Second, individuals who report higher levels of SDO and RWA are less likely to be assigned to the Feminist Group. These results are in line with previous research (e.g., Figueiredo et al., 2017) and hint that being a feminist is part of a larger set of egalitarian beliefs, beyond gender relations.

We acknowledge some limitations of this work. This study was part of a bigger survey where many other variables were asked to participants. This might reduce the quality of their responses. In addition, the measurement used to assess gender stereotypes might present certain flaws. When participants answered to what extent some traits are characteristic of a typical man and woman, they might be responding not with regards to their stereotypic perceptions, but based on how men and women actually are—due to differing education, social expectations, etc. Many gender stereotype measurements present this limitation, thus novel and appropriate measures need to be implemented. Furthermore, we are unable to study causal relationships among the variables of interest; future studies might therefore focus on the specific impact of certain variables on other related factors.

In conclusion, these results show that although we can differentiate groups of individuals according to their gender norms, traditional gender stereotypes are highly prevalent for all of them, even for those who report low levels of sexism and high support for the feminist movement. In addition, this shows that a large part of the Chilean population might be supporting feminism on the outside, but at the same time present sexist attitudes. These attitudes contribute to hindering social justice for women. Thus, in order to reach equality, top-down changes might not be enough. New gender norms should be developed from the bottom-up; norms aimed at fostering equality between men and women in daily contexts.

## Data Availability Statement

The raw data supporting the conclusions of this article will be made available by the authors, without undue reservation.

## Ethics Statement

The studies involving human participants were reviewed and approved by Comité Ético Científico en Ciencias Sociales, Artes y Humanidades. Pontificia Universidad Católica de Chile. The patients/participants provided their written informed consent to participate in this study.

## Author Contributions

GJ-M, HC, BA, and RG designed the study. BA and CC analyzed the data. GJ-M drafted the first version of the paper, HC, BA, CC, and RG participated in the writing of the posterior versions of the manuscript. All authors contributed to the article and approved the submitted version.

## Funding

This research was supported by the research grant ANID/FONDECYT 11191148, and by the Center for Social Conflict and Cohesion Studies (ANID/FONDAP 15130009).

## Conflict of Interest

The authors declare that the research was conducted in the absence of any commercial or financial relationships that could be construed as a potential conflict of interest.

## Publisher’s Note

All claims expressed in this article are solely those of the authors and do not necessarily represent those of their affiliated organizations, or those of the publisher, the editors and the reviewers. Any product that may be evaluated in this article, or claim that may be made by its manufacturer, is not guaranteed or endorsed by the publisher.
