# Supplementary material for: Is Support for Feminism Enough for Change? How Sexism and Gender Stereotypes Might Hinder Gender Justice
Source: Front Psychol. 2022 Jul 12;13:912941. doi: 10.3389/fpsyg.2022.912941 (PMC9315204; doi:10.3389/fpsyg.2022.912941)
Supplement: Supplementary file 1 [file Data_Sheet_1.docx]

**Supplementary Material**

**Model fit solutions**

**Table S1**

*Model fit solutions between one and four profiles*

|  | **AIC** | **BIC** | **aBIC** | **LMR** | **Entropy** | **Class proportions** |
| --- | --- | --- | --- | --- | --- | --- |
| 1-class | 5596.018 | 5637.962 | 5606.222 |  |  | 490 |
| 2-class | 5508.406 | 5575.516 | 5524.733 | 97.002 | 0.666 | 106, 384 |
| 3-class | 5426.304 | 5518.581 | 5448.753 | 91.636** | 0.757 | 35, 90, 365 |
| 4-class | 5376.11 | 5493.553 | 5404.681 | 60.565 | 0.765 | 43, 416, 99, 32 |

**Questionnaire items**

***Hostile sexism***

How much do you agree or disagree with each statement?

- In the name of equality, many women try to get certain privileges
- Feminists not seeking more power than men
- Women exaggerate problems they have at work
- Women seek to gain power by getting control over men

***Benevolent sexism***

How much do you agree or disagree with each statement?

- No matter how accomplished he is, a man is not truly complete as a person unless he has the love of a woman
- Every man ought to have a woman whom he adores
- A woman is incomplete without a man by her side
- Women, as compared to men, tend to have a more refined sense of culture and good taste

***Support feminist movement***

How much do you agree or disagree with each statement?

- I think the feminist movement is necessary today
- I value the feminist movement positively
- I agree with the feminist movement demands

***Traditional gender stereotypes***

Please think about what characterizes a typical man in our society. Read the list of characteristics that I will show you and indicate if you consider it to be very uncharacteristic, somewhat characteristic, or very characteristic of a typical man.

- Ambition
- Superior intelligence
- Kindness
- Energetic
- Cooperation
- Good listener
- Self-confidence
- Honest
- Independence

Please think about what characterizes a typical woman in our society. Read the list of characteristics that I will show you and indicate if you consider it to be very uncharacteristic, somewhat characteristic or very characteristic of a typical woman.

- Ambition
- Superior intelligence
- Kindness
- Energetic
- Cooperation
- Good listener
- Self-confidence
- Honest
- Independence

***SDO***

How much do you agree or disagree with each statement?

- In an ideal society, some groups should be on top and others should be on the bottom.
- Some groups of people are simply inferior to other groups.
- We should do what we can to equalize conditions for different groups ®
- We should work to give all groups an equal chance to succeed ®

***RWA***

How much do you agree or disagree with each statement?

- Instead of so much concern for the rights of the people, what this country needs is order
- Obedience and respect for authority are the most important virtues children should learn
- What our country needs is a strong authority with the determination to get us on the right path.
- The key to a good life is obedience and discipline.
- Abandoning traditions will have terrible consequences in the future
- Instead of permanently questioning the foundations of our society, in the long run it is better to be someone who is correct and who respects the rules.

**Factor loadings for Traditional gender stereotypes (masculine stereotypes)**

**Factor loadings for Traditional gender stereotypes (femenine stereotypes)**

**Table S2**

*Multinomial logistic regression predicting profile membership using the sexist group as the reference group.*

|  | Feminist | Inconsistent | Moderate |
| --- | --- | --- | --- |
| Perception of inequality | 0.46*** | 0.07 | -0.07 |
| SDO | -2.09*** | 0.28 | -0.10 |
| RWA | -2.13*** | -0.13 | -1.55* |

* p < .05 ** p < .01 *** p < .001.

**Table S3**

*Multinomial logistic regression predicting profile membership using the moderate group as the reference group.*

|  | Sexist | Inconsistent | Feminist |
| --- | --- | --- | --- |
| Perception of inequality | 0.07 | 0.14 | 0.52*** |
| SDO | 0.10 | 0.38 | -1.99** |
| RWA | 1.55* | 1.42** | -0.58 |

* p < .05 ** p < .01 *** p < .001.

**Table S4**

*Multinomial logistic regression predicting profile membership using the inconsistent group as the reference group.*

|  | Sexist | Feminist | Moderate |
| --- | --- | --- | --- |
| Perception of inequality | -0.07 | 0.39** | -0.14 |
| SDO | -0.28 | -2.37*** | -0.38 |
| RWA | 0.131 | -2.00*** | -1.42** |

* p < .05 ** p < .01 *** p < .001.
